# Relationship between carina size and sternum morphology in birds reflects physical constraints of body size and flight style

**DOI:** 10.1111/joa.70190

**Published:** 2026-06-16

**Authors:** D. C. Deeming

**Affiliations:** ^1^ Joseph Banks Laboratories, School of Natural Sciences University of Lincoln Lincoln UK

**Keywords:** flight style, keel area, sternum area, sternum morphology, taxonomy

## Abstract

The avian sternum is the largest bone in the body to accommodate the large muscles required for flight. Previous analysis showed that variation in keel and sternum morphology only weakly reflected body size and flight style and implied that variation in caudal metasternum morphology was associated with expansion of bony trabeculae in some species. This study used area measurements to explore the idea that, to minimise body mass, variation in keel size was inversely correlated with variation in the shape of the metasternum. Data for sternum dimensions and area were collected from digital images of pectoral girdles and the sternum in articulation of 62 species representing 10 different orders. The keel area was expressed as a proportion of the total sternum area, and the area of the sternum from the ventral aspect occupied by bone was expressed as a proportion of the assumed maximum possible bone area. Phylogenetically controlled linear modelling explored the effects of body mass, order, sternum type and flight style. Sternum area and keel area exhibited isometric relationships with body mass which had different intercepts for each order. Proportional keel area was inversely related to body mass in some but not all orders. The proportional bone area exhibited a positive relationship with body mass and there was a significant effect of order. Proportional keel area exhibited a negative relationship with the proportional bone area. Both sternum type and flight style significantly affected proportional keel area and the proportional bone area. It was concluded that to save body mass, an increase in bone mass associated with the development of a keel has been mitigated by bone not developing (rather than extension of existing bone) in the caudal metasternum. Such patterns would also explain observed variation in sternum morphology in Mesozoic birds.

## Graphical abstract

Sternum morphology varies among birds and is a function of body mass and order. The area of the sternum is inversely related to the area of the keel suggesting that the increase in bone mass associated with a large keel has been mitigated by a reduction in bone mass of the sternum.

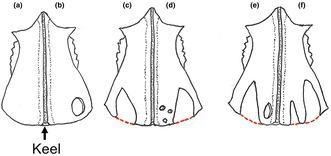

## 1 INTRODUCTION

The avian sternum is the largest bone in the body playing a key role in powered flight because it serves as the point of origin for the flight muscles that drive movement of the wings (Ritchison, 2023). The functional surface area for muscle attachment of the sternum has been increased by evolution of a keel (carina) that projects ventrally along the midline and varies in size and shape among species (Gündemir et al., 2023; Lowi‐Merri et al., 2021), a trait that has been useful in the taxonomy of birds (Heiderdinger & Ames, 1967). In addition, the caudal edge of the metasternum can range from being entire or having a fenestra in the caudal part of the bone or having one or two notches that extend cranially (Heiderdinger & Ames, 1967). Recently, there has been a greater appreciation of the functional effects of variation in sternum morphology, which were explored using geometric morphometric techniques in relation to factors such as body mass and flight style (Lowi‐Merri et al., 2021). It was concluded that although significant, correlations between shape in morphospace and locomotion were weak and it was likely that other factors were involved in shaping the sternum.

Lowi‐Merri et al. (2021) showed that sternum shape in morphospace was a function of body size with larger birds having relatively shallower keels than smaller birds, but their illustrations showed that there was considerable variation across species in both the size of the keel and the complexity of the metasternum. This variation may have important functional consequences in two key areas.

Firstly, Deeming and Ferrari Da Silva (2025) showed that the length of the coracoid correlated with wingspan, and they suggested that there could also be variation in sternum dimensions that could affect the length of the flight muscles and hence flight patterns. Therefore, variation in the depth of the sternum and/or the keel may have implications for the maximum length of the *m. pectoralis* and *m. supracoracoideus*. For instance, assuming the coracoid does not change size, the ventral edge of a deep keel (or long sternum) will be further away from the shoulder joint and the muscles that attach here will be longer. Size of the stroke amplitude angle shows negative relationships with body mass, wingspan and the length of the forelimb skeleton (Nudds et al., 2004). One consequence of an enlarged keel that has yet to be considered is that variation among species in bone area associated with a keel could affect the total size of the sternum, which, being the largest bone in the body, could influence the mass of the bird. In addition, the larger area of bone for the origin of the *m. pectoralis* could also affect body mass by an increased muscle mass.

**FIGURE 1:**
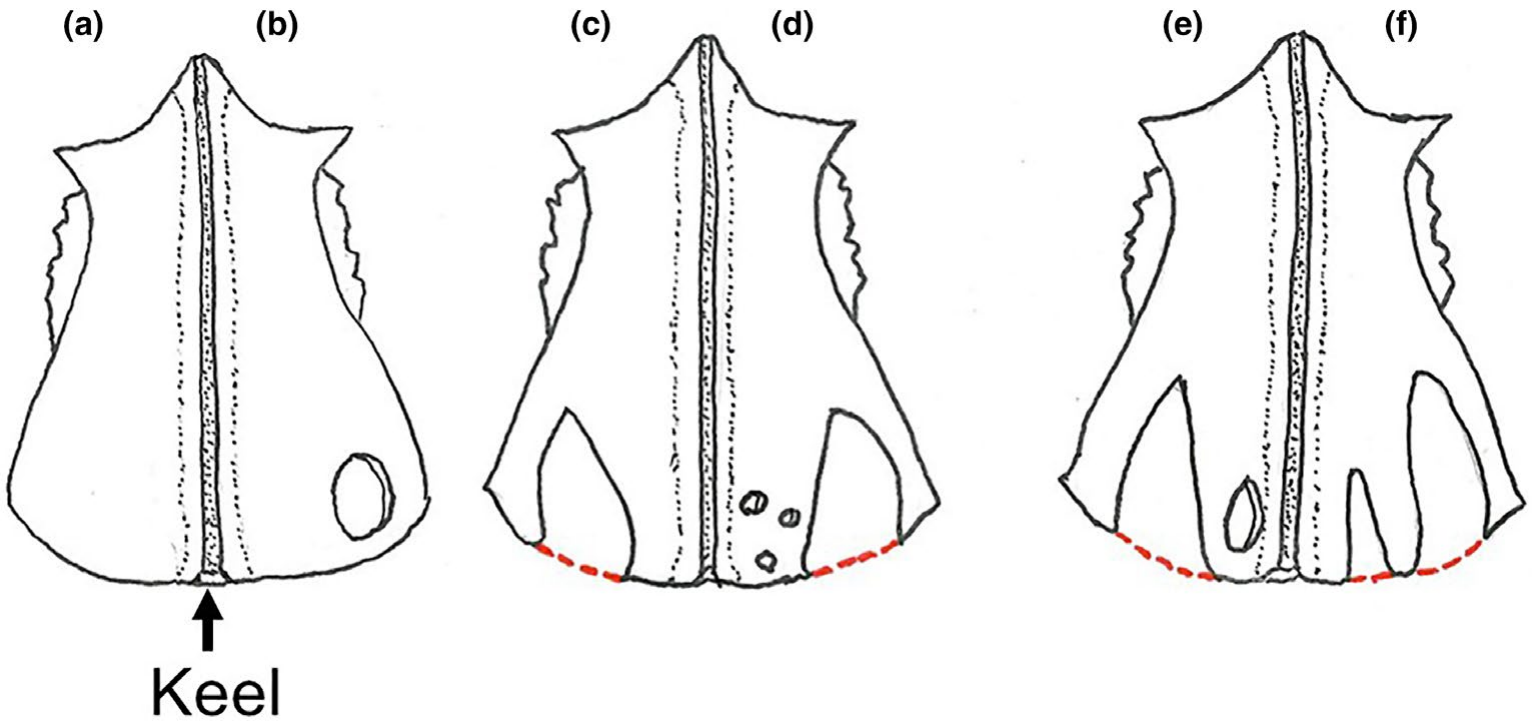
Ventral views of an avian sternum showing the differing degrees of bone loss from the caudal part of the metasternum. (a) No bone loss; (b) Lateral fenestra; (c) Lateral sternal notch; (d) Lateral sternal notch with small medial fenestrae; (e) Lateral sternal notch with large medial fenestra; (f) Lateral and medial sternal notchs. Adapted from Heiderdinger and Ames (1967). Red dashed lines indicate the line assumed to define the caudal limit of the sternal notch.

Secondly, there is variation in the size and number of the sternal fenestra and notches at the caudal end of the metasternum (Heiderdinger & Ames, 1967; Figure 1). Lowi‐Merri et al. (2021) recognised that size and degree of fusion of the caudal trabeculae (processes described by Heiderdinger & Ames, 1967) would have implications for areas for muscle attachment. It was implied that variation in morphology reflected longer trabeculae meaning that the caudal metasternum was being supplemented by bone. However, a sternal fenestra is a place where bone is missing, and a sternal notch is just a fenestra enlarged by the absence of bone caudally. Perhaps any increase in bone mass associated with evolution of a large, deep carina could have been mitigated for by a reduction in bone area in the metasternum? Could the ancestral state for the sternum with a shallow keel be a fully ossified caudal metasternum that extends across the width of the bone? In smaller species, an increase in keel area (and bone mass) in order to increase stroke amplitude angle (Deeming & Ferrari Da Silva, 2025) would mean that to minimise the mass of the sternum as a whole, there would have been a decrease in bone volume of the metasternum by increasingly larger or more numerous sternal fenestra or notches. Increased complexity of metasternum morphology has also been noted in Mesozoic birds with more complex metasternum morphology in smaller and more derived species (O'Connor et al., 2015; Zheng et al., 2012, 2014).

Using an alternative approach to the geometric morphometrics used by Lowi‐Merri et al. (2021), in this study described here measurements were taken from digital images of articulated sternums and pectoral girdles to allow for determination of a variety of variables. First, total area of the sternum and keel were measured and the proportion of the total surface area of the sternum associated with the area of the keel was calculated. Second, the proportion of the maximum sternum area, as observed from the ventral perspective that was associated with bone, was calculated. Observations of scaling of muscular and skeletal elements of the flight apparatus in birds have demonstrated that morphological variation is related to avian order, that is, for any given body mass of bird, the mass of the *m. supracoracoideus* or length of the coracoid length are significantly affected by order (Deeming, 2023; Deeming & Ferrari Da Silva, 2025).

These variables were used to explore the following hypotheses. Firstly, there would be isometric scaling with body mass for sternum length, and sternum and keel areas, and there would be an effect of order on the magnitude of the areas for any given body mass. Second, if larger stroke amplitude angles in smaller birds are associated with deeper keels, then the relative size of the keel should be inversely related with body size and would be related to order. Thirdly, the proportional area of sternum bone observed from a ventral perspective would be reflected in sternum type with a decrease in bone area as the size and number of fenestra and notches in the metasternum increase. Finally, if large notches reflected a decrease in bone area, then a negative relationship with relative keel area is predicted. The analyses were also repeated to determine the potential effects of flight style or sternum morphology.

## 2 MATERIALS AND METHODS

### 2.1 Data sources

**FIGURE 2:**
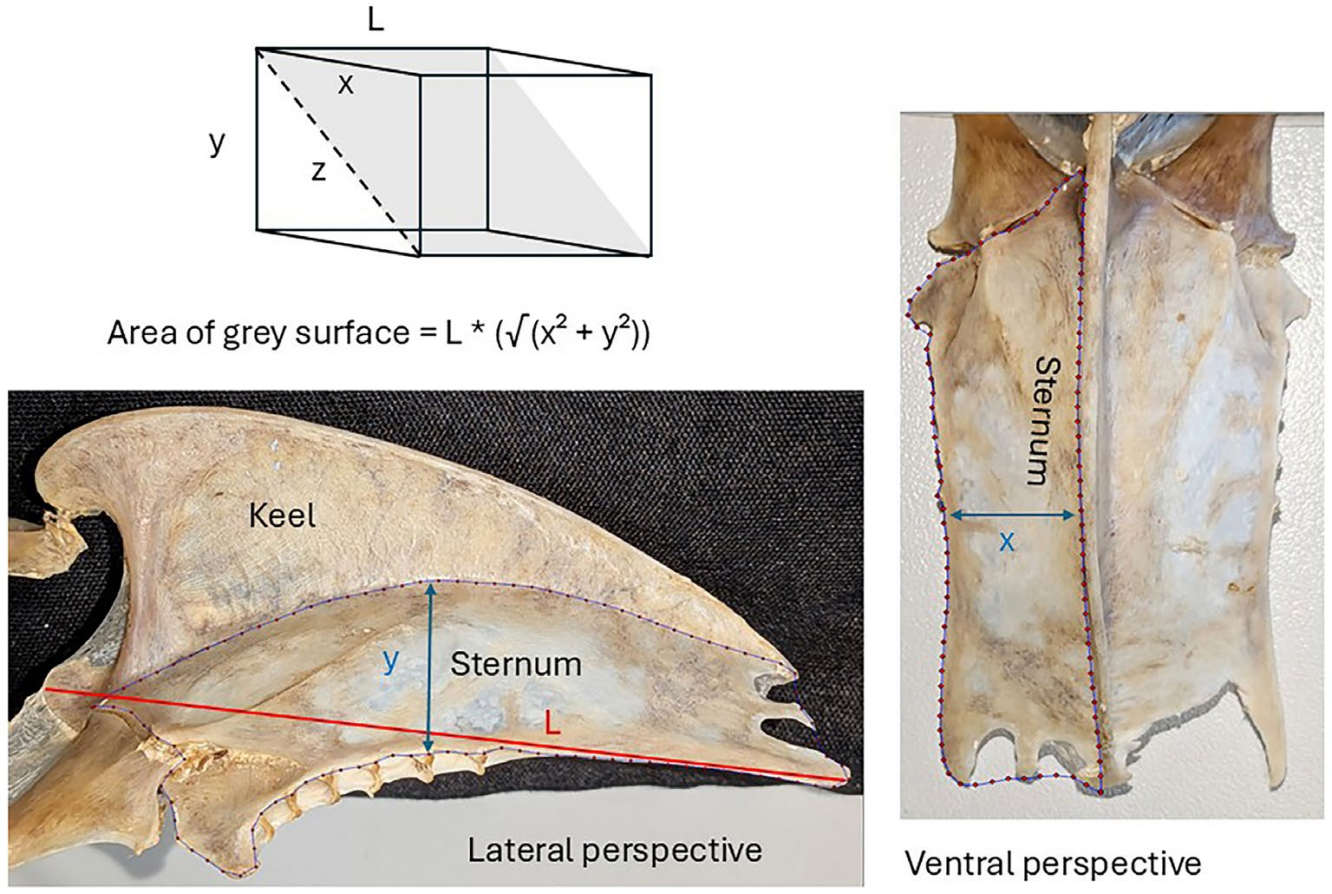
Illustration of the area of the sternum (*Larus argentatus*) outlined in ImageJ to measure sternum area from the lateral and ventral perspectives. Sketch in top left illustrates the principle of how an area of the three‐dimensional ventral surface of the structure was calculated (see text for more details). *L* = maximum length of the sternum, *y* = depth of the sternum from a lateral perspective, *x* = width of the sternum from a ventral perspective, *z* = distance between the base of the carina and the lateral edge of the sternum determined using Pythagoras' theorem (*z* = √(*x* ^2^ + *y* ^2^)).

Data were collected from digital images of the skeletal elements of the pectoral girdle and sternum taken with a Pentax K50 dSLR camera fitted with a 50 mm Sigma macro lens. Specimens were photographed at the Natural History Museum, Tring, United Kingdom, and were selected on the basis that the pectoral girdle, coracoid and sternum were intact and in natural articulation. If possible, species with a range of body masses were selected. Images with taken from the lateral and ventral perspectives (Figure 2). Images were taken of skeletons of 62 species representing 10 different orders, with between three and ten representative species in each order (Table S1). Mean body mass values for the species were used from Dunning Jr. (2008). Each species was allocated to a sternum type (A, B, C etc.) as illustrated in Figure 1 (Heiderdinger & Ames, 1967). Each species was also allocated to one of the following flight types as defined by Lowi‐Merri et al. (2021): burst flier (BF), continuous flapping (CF), flap‐gliding (FG) and soaring (SO). The distribution of the species with respect to mean body mass, order, sternum type and flight style is shown in Table S1.

### 2.2 Data collection

Due to the three‐dimensional nature of the sternum, its surface area (in mm^2^ in all cases) was calculated as follows:(see Figure 2). Firstly, using the line tool in ImageJ (Schneider et al., 2012), calibrated using an in‐image scale, the maximum length of the sternum (L, in mm) was determined from the cranial to caudal tips (Figure 2). Secondly, the edge of the sternum as viewed from the lateral perspective was traced using the polygon tool to determine its area. The average depth (‘y’ in mm) of the sternum was calculated by dividing the lateral area by the sternum length. Thirdly, the maximum area of the sternum from the ventral perspective was recorded by tracing around the edge of the bone. If sternal notches were present a straight line was drawn across the two caudal points of the metasternal trabeculae (Figure 2). Thereafter, the area of each sternal notch, or if present the sternal fenestra, was determined in ImageJ and the areas where bone was missing were combined and this value subtracted from the area of the maximum possible sternum area from the ventral perspective (Figures 1 & 2). Following the method used for the lateral perspective, the mean width of the bone was calculated (‘x’ in mm) from the area and the sternum length. Pythagorean geometry was then used to determine the average length of the angled surface of the sternum (‘z’ in mm), which was then multiplied by the length of the sternum to give the angled surface area of the sternum (in mm^2^; Figure 2). Values were determined for the one side of the sternum and doubled to get the total surface areas of the keel and the sternum, and these values were combined to give the total sternum area (sternum + keel). For the sternum illustrated in Figure 2, the estimated surface area was 2014 mm^2^ and the area determined from measurements using callipers (length × average of maximum and minimum width) was 1895 mm^2^ (94% of estimated area). The proportion of the total sternum area occupied by the keel (KA/TSA) was determined by dividing total keel area (KA) by the total sternum area (TSA). Finally, the proportion of the ventral surface occupied by bone (PropBone) was determined by dividing the maximum possible surface of the ventral surface of the sternum by the area of the ventral surface that was occupied by bone.

### 2.3 Statistical analysis

All area data were log_10_‐transformed prior to analysis, whereas proportional data were logit‐transformed (Warton & Hui, 2011), to obtain residuals that were normally distributed and homoscedastic. Analysis was carried out using phylogenetically controlled general linear modelling (pglm) performed in R (R Core Development Team, 2020) using the packages *ape* (Paradis et al., 2004), *mvtnorm* (Genz & Bretz, 2009) and *MASS* (Venables & Ripley, 2002) as used previously (e.g. Deeming, 2022, 2023). Phylogenetic relatedness was controlled to account for non‐independence of data points and used a time‐calibrated phylogeny pruned in R from a phylogenetic tree that included all bird species (Jetz et al., 2012).

Phylogenetically controlled generalised linear regression analysis tested the allometric relationships between body mass as a covariate and order as a fixed factor, respectively, for (1) L, (2) TSA, (3) KA, (4) KA/TSA and (5) PropBone. In addition, analysis also tested the relationship between PropBone and KA/TSA as a covariate and order as a fixed factor. Further analyses tested the relationships between KA/TSA and PropBone using body mass as a covariate and either sternum or type of flight style as a fixed factor.

Initial models included an interaction term but if this was shown to be non‐significant the model was simplified and run again. All models incorporated phylogeny as a random effect to deal with non‐independence among species. Model quality was reported as the coefficient of determination (*R* ^2^) and the phylogenetic signal (lambda, *λ*) was calculated with values ranging from zero to one representing no covariance in the residuals, and the observed covariance in residuals was expected under a Brownian motion model of trait evolution, respectively (Freckleton et al., 2002). Departure from isometry for L, TSA and KA was tested by using a one‐sample *t*‐test to compare the slope of the regression estimate, with its standard error, against an expected isometric slope of 0.333 for length and 0.667 for body mass (Bailey, 1981).

## 3 RESULTS

### 3.1 Effects of taxonomy

Sternum length exhibited a positive relationship with body mass with some orders, like the Anseriformes, having longer sternums for a given body mass than others, for example the Strigiformes (Figure S1), but there was no significant interaction between body mass and order (*F*_9,42_ = 0.91, *p* = 0.527). In the simplified model, order was a significant fixed factor and body mass (LogBM) was a significant covariate (Table 1). The slope of the relationship between L and body mass (0.329 ± 0.021[se]) was not significantly different from an isometric slope of 0.333 (*t*_61_ = −0.22, *p* = 0.830).

**TABLE 1:** Results of phylogenetically controlled analyses of relationships between various metrics of sternum and keel area, keel area as a proportion of the total sternum area (KA/TSA), and the proportion of the ventral view of the sternum that is occupied by bone (PropBone).

Relationship	Covariate (*p*‐value) [DF = 1,51]	Order (*p*‐value) [DF = 9,51]	Interaction	*R* ^2^	*λ*	Slope (SE)
Sternum length versus body mass	754.21 (<0.001)	7.06 (<0.001)	—	0.941	<0.0001	0.329^c^ (0.021)
Total sternum area versus body mass	956.54 (<0.001)	8.85 (<0.001)	—	0.953	<0.0001	0.634^c^ (0.035)
Keel area versus body mass	517.29 (<0.001)	11.30 (<0.001)	—	0.924	<0.0001	0.582^d^ (0.042)
KA/TSA versus body mass	48.31^a^ (<0.001)	12.46^b^ (<0.001)	2.27^b^	0.812	<0.0001	—
PropBone versus body mass	72.21 (<0.001)	24.29 (<0.001)	—	0.851	<0.0001	0.029 (0.011)
PropBone versus KA/TSA	119.15 (<0.001)	16.36 (<0.001)	—	0.839	<0.0001	−0.175 (0.100)

*Note*: Prior to analysis, data for body mass, sternum area and keel area were log transformed and proportion data were logit‐transformed. Values show *F*‐value with associated *p*‐values, coefficient of determination (*R* ^2^) and lambda (*λ*) values for the model. Interaction terms are only reported if they are significant (otherwise reported in text). Values (and SE) for the estimate of the slope for relationships with no significant interaction are also shown.

^a^ DF = 1,42.

^b^ DF = 9,42.

^c^ Slope not significantly different from isometry.

^d^ Slope significantly different from isometry at *p* < 0.05.

**FIGURE 3:**
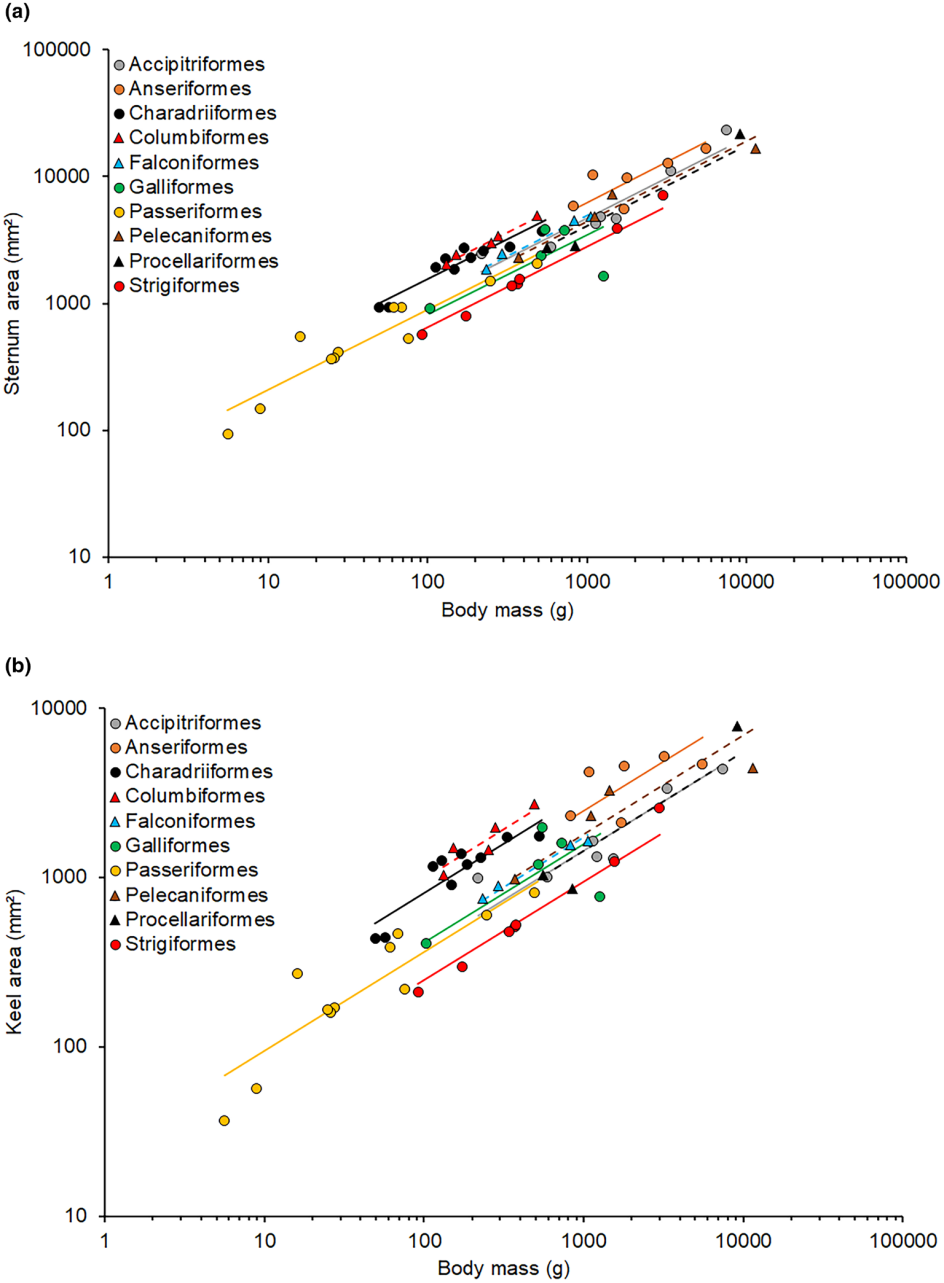
Relationships between body mass and (a) sternum area and (b) keel area for 10 orders. Lines indicate phylogenetically controlled relationships calculated in R for each order (see text).

There was a positive relationship between body mass and total sternum area with the various orders exhibiting different intercepts (Figure 3(a)) although the interaction term between TSA and order was not significant (*F*_9,42_ = 0.74, *p* = 0.674). For the simplified model, LogBM was a significant covariate, and order was a significant fixed factor (Table 1). The slope of the relationship (0.634 ± 0.035) was not significantly different from an isometric slope of 0.667 (*t*_61_ = −0.94, *p* = 0.351).

There was also a positive relationship between body mass and KA, and orders seemed to exhibit greater differences in intercepts than for the total sternum area (Figure 3(b)). The interaction between body mass and order was not significant (*F*_9,42_ = 1.05, *p* = 0.419) and in the simplified model both LogBM and order significantly affected KA (Table 1). Like TSA, the slope of the relationship between body mass and KA (0.581 ± 0.142) was not significantly different from an isometric slope of 0.667 (*t*_61_ = −0.59, *p* = 0.557).

**FIGURE 4:**
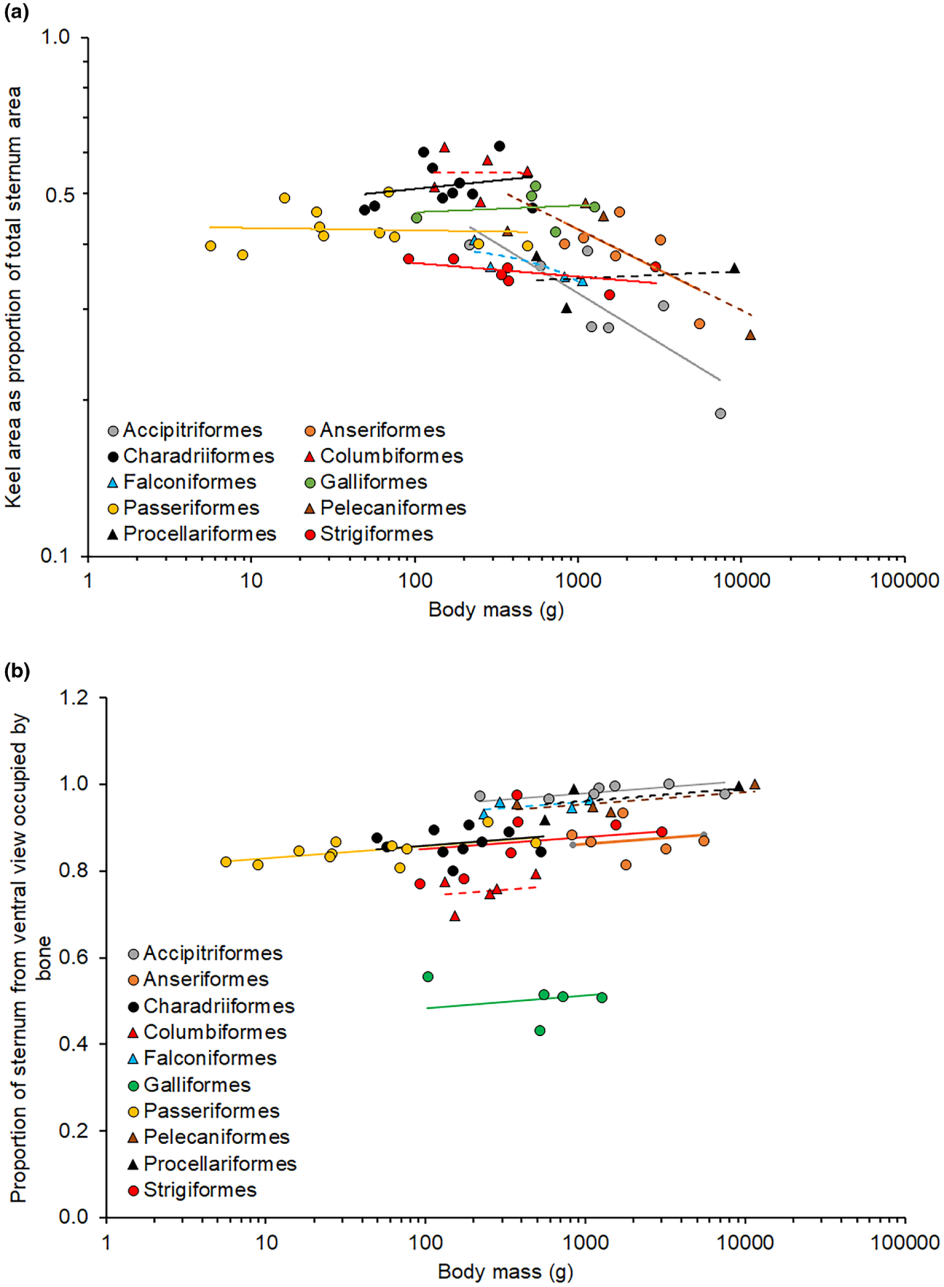
Relationships between body mass and (a) keel area as a proportion of the total sternum area (KA/TSA), and (b) the proportion of the ventral view of the sternum that is occupied by bone (PropBone) for 10 orders. Lines indicate phylogenetically controlled relationships calculated in R for each order (see text).

When keel area was expressed as a proportion of the total sternum area (KA/TSA) there was a generally negative relationship with body mass, but order seemed to be important because, for instance, KA/TSA was high for Charadriiformes but lower for Strigiformes (Figure 4(a)). The interaction term for the model for the KA/TSA ratio was marginally significant, body mass was a highly significant covariate, as was order (Table 1). The model explained a high proportion of the variation in the data and the phylogenetic signal (*λ*) was very low (Table 1).

There was a shallow positive relationship between body mass and the proportion of the sternum (as viewed from the ventral perspective) associated with bone (PropBone), with different orders exhibiting quite different patterns (Figure 4(b)). In particular, the Accipitriformes had the highest values with little or no apparent loss of bone. At the other extreme, the Columbiformes had low values where there was around 25% loss of bone area, although the Galliformes had considerably lower values, with only around 50% of the ventral sternum area being associated with bone. The interaction term for the model comparing LogBM and order was not significant (*F*_9,42_ = 1.42, *p* = 0.158). Both order and LogBM were significant factors affecting PropBone (Table 1).

**FIGURE 5:**
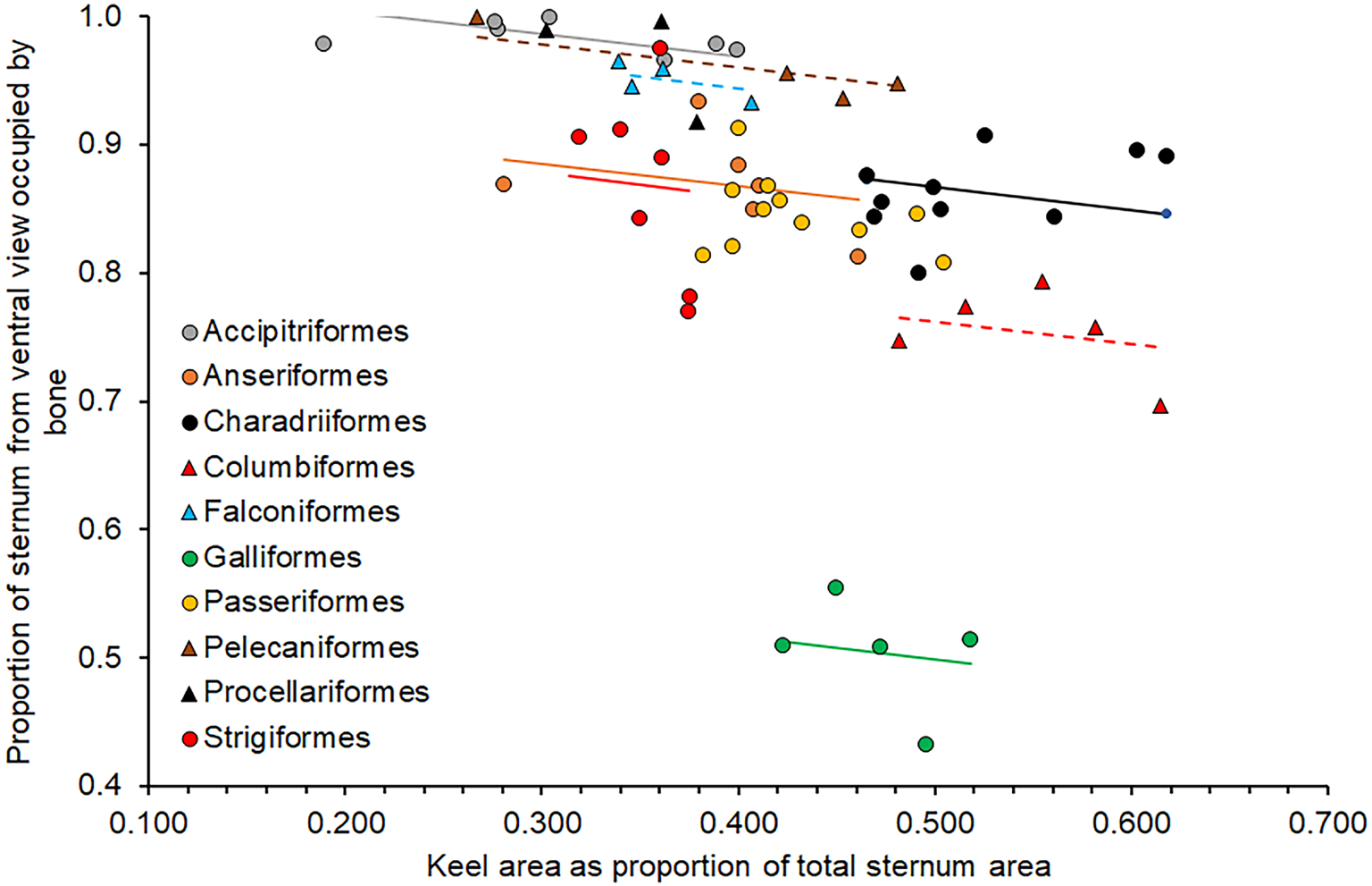
Relationships between the keel area as a proportion of the total sternum area (KA/TSA) and the proportion of the ventral view of the sternum that is occupied by bone (PropBone) for 10 orders. Lines indicate phylogenetically controlled relationships calculated in R for each order (see text).

The relationship between Propbone and KA/TSA ratio had a relatively shallow negative gradient (Figure 5). Order seemed to be important in determining the intercept with species of the Charadriiformes having comparable KA/TSA ratios as the Columbiformes, but the latter had much smaller proportions of bone (Figure 5). The full model for KA/TSA showed that the interaction term between LogBM and order was not significant (*F*_9,42_ = 0.90, *p* = 0.537). By contrast, in the simplified model, body mass and order were both significant determinants of the proportion of bone area (Table 1).

### 3.2 Effects of sternum type

Body masses were generally larger in sternum types with fewer notches or fenestrae in the metasternum (Figure S2). As shown previously, KA/TSA declined with body mass, but the values were also lower for those species where the sternum lacked any caudal notches (Figure S3A). KA/TSA did not exhibit a significant interaction between body mass and sternum type (*F*_4,52_ = 0.35, *p* = 0.787). In the simplified model, LogBM was a significant covariate, but despite observed differences, sternum type did not significantly affect KA/TSA (Table 2).

**TABLE 2:** Results of phylogenetically controlled analyses of relationships between body mass and keel area as a proportion of the total sternum area (KA/TSA), and the proportion of the ventral view of the sternum that is occupied by bone (PropBone).

Relationship	Covariate (*p*‐value) [DF = 1,56]	Sternum type style (*p*‐value) [DF = 4,56]	*R* ^2^	*λ*	Covariate (*p*‐value) [DF = 1,57]	Flight style (*p*‐value) [DF = 3,57]	*R* ^2^	*λ*
KA/TSA versus body mass	12.96 (0.001)	1.78 (0.146)	0.264	0.858	18.47 (<0.001)	5.25 (0.003)	0.375	0.752
PropBone versus body mass	38.61 (<0.001)	15.55 (<0.001)	0.643	0.958	90.65 (<0.001)	70.73 (<0.001)	0.842	0.026

*Note*: Prior to analysis, data for body mass were log transformed and proportion data were logit transformed. Values show *F*‐value with associated *p*‐values, coefficient of determination (*R* ^2^) and lambda (*λ*) values for the model. Interaction terms are only reported if they are significant (reported in text).

Bone area in the ventral aspect (PropBone) increased with body mass, with the species lacking sternal notches again having the highest values (Figure S3B). PropBone did not exhibit a significant interaction between LogBM and sternum type (*F*_4,52_ = 1.00, *p* = 0.417). The simplified model showed that both LogBM and sternum type were highly significant determinants of PropBone (Table 2).

### 3.3 Effects of flight style

Body masses were generally larger in birds exhibiting soaring flight and lowest for birds with continuous flapping (Figure S2). Soaring birds had low values for KA/TSA whereas the highest values were for species with continuous flapping or burst flight (Figure S3C). There was a non‐significant interaction term between LogBM and flight style for KA/TSA (*F*_3,54_ = 0.35, *p* = 0.787). Both LogBM and flight style in the simplified model had highly significant effects on KA/TSA (Table 2).

PropBone was highest for species that soar or flap‐glide, but considerably lower for species, that is, Galliformes, with burst flight (Figure S3D). PropBone had a non‐significant interaction between LogBM and flight style (F_3,54_ = 0.35, *p* = 0.787), but in the simplified model both had highly significant effects (Table 2).

## 4 DISCUSSION

Sternum morphology was related to body size, with larger birds having a shallow keel and a wide metasternum lacking any notches or fenestra. However, as bird size decreased, the depth of the keel increased and the proportion of bone of the sternum progressively decreased, even though the total area of the sternum plus keel scaled isometrically. This pattern was also associated with flight style and with sternum type, but this may have reflected a covariance of these criteria with order. For instance, large, soaring birds of prey (Accipitriformes) had relatively shallow keels on type A sternums (no notches or fenestra), whereas small passerines (Passeriformes), that need to continuously flap their wings when flying, had type F sternums (two extensive notches) with relatively deep keels. There were, however, no birds of prey with type F sterna and no passerines with type A sternums. There was a significant negative relationship between KA/TSA and PropBone, with significant effects of order, sternum type and flight style.

The size of the sternum and the keel relative to thoracic length is a function of mode of locomotion (Bickley & Logan, 2014; Feneck et al., 2021), although Lowi‐Merri et al. (2021) showed that there were significant but weak associations between variation in sternal morphology and body size, and with flight style, which has been broadly confirmed here. The present study has demonstrated that there is a link between keel size and the complexity of the caudal metasternum that was not perhaps immediately obvious from analysis employing geometric morphometrics that analysed sternum shape as a whole (Lowi‐Merri et al., 2021). Here total sternum and keel areas exhibited isometric relationships with body mass, but the proportion of the area associated with the keel was strongly associated with sternum type. In the species studied, complex caudal sternal morphology was associated with species that are small, have relatively deep keels and tend to continuously flap to maintain a large stroke amplitude angle (Nudds et al., 2004). By contrast, large species have smaller stroke amplitude angles, shallower keels and no complex metasternal shape, and large flightless ratites have type A sternums (no notches or fenestra) and no keel (Kassem et al., 2024; Lowi‐Merri et al., 2021).

The differences in caudal morphology of the sternum were recognised by Lowi‐Merri et al. (2021), but these shapes were interpreted as being associated with the relative expansion of the sternal trabeculae depending on flight style. There is, however, a more parsimonious explanation of this variation, which has been confirmed by the relationship between KA/TSA and PropBone. A deep keel comprises a lot of bone, which will add mass to the already large sternum. However, there was an isometric relationship between sternum area and body mass that suggests that the increase in keel area has been mitigated by a reduction in the size of the metasternum. It is acknowledged that the slope of the relationship in Figure 5 is not particularly strong, but it is a significant negative relationship as predicted. This suggests that it is an absence of bone that creates the fenestrae and sternal notches, rather than extension of body trabeculae as suggested by Lowi‐Merri et al. (2021).

During embryonic development the sternum is formed by the union of lateral sternal plates of cartilage that are created by mesenchymal condensation (Romanoff, 1960). The three‐dimensional shape of the bone arises from fusion of the lateral plates along the midline, and the keel is formed by migration of cells ventrally from the sternal perichondrium (Romanoff, 1960). The budgerigar (*Melopsittacus undulatus*) has one caudal fenestra on either side of the keel, which is formed by elongation of the posterior lateral processes curving backwards to fuse with the corpus sterni leaving spaces surrounded by cartilage (Fell, 1939). Therefore, in one species at least, a sternal foramen seems to be caused by reduced cartilage deposition during development, which subsequently becomes ossified (Nakane & Tsudzuki, 1999). Whether this pattern is applicable to all bird species is not known and is worthy of further investigation. Sternum development seems to be related to the T‐box transcription factor *Tbx5*, and lack of expression has been associated with defects in sternum morphology (Feneck et al., 2021). Perhaps, variation in caudal sternum morphology among birds is a function of control of cartilage and then bone deposition by Tbx5 expression during development?

Small birds with deep keels needed to drive large stoke amplitude angles (Nudds et al., 2004) seem to have maintained a total sternum area by reducing the area of the bone in the caudal metasternum. Total skeletal mass in birds exhibits positive allometry (Martin‐Silverstone et al., 2015; Prange et al., 1979), so smaller birds have relatively lighter skeletons (5.3% of body mass for a 50 g bird compared with 6.2% for a 500 g bird and 7.3% for a 5 kg bird). A reduced sternum area has presumably helped mitigate any increase in body mass associated with bone forming a larger keel. An extreme example of this is shown in the Galliformes, that have disproportionately large supracoracoideus muscles (Deeming, 2023) to power strong, rapid near‐vertical take‐off bursts of flight to escape from predators (Pennycuick et al., 1994). Mass would be of a premium for such species because any excessive weight would have limited the efficacy of burst flight. Therefore, extensive sternal notches mean that there is relatively little bone in the lateral sternum, which consists of rib‐like trabeculae (see illustrations of the sternum of Lady Amherst's pheasant [*Chryodophus amerherstiae*] in Wen & Yang, 1991 and Zheng et al., 2012). Columbiformes also have excellent vertical acceleration (Dial, 1992) associated with a relatively large *m. supracoracoideus* (Deeming, 2023). Whilst they may have been classified here as species with continuous flapping, in this study, these species were relatively heavy (132–490 g) and had the next lowest PropBone values in the dataset (<0.8) because of a lateral notch and a medial fenestra in the metasternum. The relationship in Figure 4(a) suggests that very small hummingbirds would have large KA/TSA values. This is not unreasonable because these species have extremely larger keels and type A sternums, which are very narrow laterally (Zusi, 2013). The possibility that hummingbirds have small sternum areas due to absence of bone across the whole bone, rather than just caudally, is worthy of further research. An interesting area for future research would be how loss of bone has impacted on the biomechanical properties of the sternum when under strain imposed by muscle contraction to power the wings.

Changes in the size and morphology of the sternum have also been recognised in the fossil record of Mesozoic birds (Lowi‐Merri et al., 2023) and, in particular, the morphology of the caudal metasternum is comparable to many extant species (Lowi‐Merri et al., 2021). In extinct species, like extant species (Lowi‐Merri et al., 2021), the shape of the caudal metasternum was considered to be the result of elongation of the trabeculae (Lowi‐Merri et al., 2025). However, this interpretation is despite the fact that lateral and medial trabeculae rarely extend far beyond the caudal edge of the sternal keel (see illustrations in Zheng et al., 2014; O'Connor et al., 2015; Mayr, 2017). In addition, the trabeculae are very short in the relatively large (up to 487 g) *Ichthyornis* from the Late Cretaceous (Lowi‐Merri et al., 2023, 2025). The interpretation of the data from the present study suggests that, rather than sternal trabeculae extending in smaller Mesozoic birds (Zheng et al., 2014), it is reduced bone deposition that has created the distinctive morphology of the caudal metasternum. A driver for such a change could have been the evolution of a larger keel, which was relatively well‐developed in the enantiornithine and ornithuromorph lineages (O'Connor et al., 2015). Loss of bone in the metasternum would make sense in this context because most enantiornithine and ornithuromorph lineages were small and probably weak flyers (Zheng et al., 2014), so any mechanism to reduce body mass and aid in flight efficacy would have been selected for. Evolution of sternal shape is considered episodic (Lowi‐Merri et al., 2025) and as body size of birds increased towards the end of the Cretaceous, then there was no need for reduced bone in the metasternum, and so *Ichthyornis* had only small sternal notches (Lowi‐Merri et al., 2023, 2025).

It is appreciated that the method to calculate sternum area in this study was dependent on certain assumptions regarding the three‐dimensional structure of the sternum, and the extent to which bone might fill a caudal sternal notch. However, the method did provide good approximations of sternum area that could be used in other comparative analyses. It would be interesting to explore whether 3D scanning of the bone offers a more accurate measure of the surface area of the sternum.

## 5 CONCLUSION

In conclusion, there is an inverse relationship between the relative size of the keel and the degree of bone coverage in the caudal metasternum. Analysis revealed that this was closely associated with body mass, with small birds being restricted to certain flight styles that were associated with complex metasternum morphology. However, contrary to previous studies, this variation in morphology is attributed to loss of bone, which mitigates the increased area of bone in the keel, and may have also applied to birds in the Mesozoic. This study supports previous comparative studies that demonstrate that flight patterns in birds are not simply a function of wing size or shape (Norberg, 1990), but reflect variation in the underlying skeleton (Akeda & Fujiwara, 2023; Deeming & Ferrari Da Silva, 2025; Gündemir et al., 2023; Lowi‐Merri et al., 2021; Serrano et al., 2020) and musculature (Deeming, 2023; Deeming & Mosto, 2026) of the sternum, pectoral girdle, and wing.

## Supporting information


**Table S1.** Number of species per order in each flight category and sternum type, together with the number of bird species in each flight category that had each type of sternum (see Figure 1).
**Figure S1.** Relationship between body mass and sternum length for 10 orders. Lines indicate phylogenetically controlled relationships for each order (see text).
**Figure S2.** Boxplots showing the median, interquartile ranges and range of values for body mass for each sternum type (left panel) and flight style (right panel).
**Figure S3.** Relationships between body mass and (A) keel area as a proportion of the total sternum area (KA/TSA), and (B) the proportion of the ventral view of the sternum that is occupied by bone (PropBone) for five different sternum shapes, and body mass and (C) KA/TSA, and (D) Propbone for four flight styles. Lines indicate phylogenetically controlled relationships calculated in R for each order (see main text).

## Data Availability

The data that support the findings of this study are available from the corresponding author upon reasonable request.
