# Supplementary material for: Relationship between carina size and sternum morphology in birds reflects physical constraints of body size and flight style
Source: J Anat. 2026 Jun 16:10.1111/joa.70190. Online ahead of print. doi: 10.1111/joa.70190 (PMC13398643; doi:10.1111/joa.70190)
Supplement: Supplementary file 1 — Table S1. Number of species per order in each flight category and sternum type, together with the number of bird species in each flight category that had each type of sternum (see Figure 1). Figure S1. Relationship between body mass and sternum length for 10 orders. Lines indicate phylogenetically controlled relationships for each order (see text). Figure S2. Boxplots showing the median, interquartile ranges and range of values for body mass for each sternum type (left panel) and flight style (right panel). Figure S3. Relationships between body mass and (A) keel area as a proportion of the total sternum area (KA/TSA), and (B) the proportion of the ventral view of the sternum that is occupied by bone (PropBone) for five different sternum shapes, and body mass and (C) KA/TSA, and (D) Propbone for four flight styles. Lines indicate phylogenetically controlled relationships calculated in R for each order (see main text). [file JOA-9999-0-s001.pdf]

Relationship between carina size and sternum morphology in birds reflects physical constraints of body size and flight style

D. C. Deeming

School of Natural Sciences, University of Lincoln, Joseph Banks Laboratories, Lincoln, LN6 7DL, UK

Correspondence: cdeeming@lincoln.ac.uk

Supplementary materials

Table S1. Number of species per order in each flight category and sternum type, together with the number of bird species in each flight category that had each type of sternum (see Figure 1).

						Sternum type					
Order	Mean body mass (± SD)	Burst flight	Continuous flapping	Flap-glide	Soaring	A	B	C	D	E	F
Accipitriformes	2208.0 (2508.6)			3	4	1	6				
Anseriformes	2358.3 (1767.8)		6				1	5			
Charadriiformes	193.6 (142.6)		10					2		1	7
Columbiformes	261.2 (142.6)		5							5	
Falconiformes	604.0 (405.9)			4			4				
Galliformes	630.6 (420.5)	5									5
Passeriformes	95.4 (44.2)		9	2				11			
Pelecaniformes	3594.3 (5226.3)			3	1	1		3			
Procellariformes	3505 (4856.2)			2	1		2				1
Strigiformes	843.0 (1066.9)		7					1		1	5

	Sternum type					
	A	B	C	D	E	F
Burst flight						5
Continuous flapping		1	17		6	13
Flap-glide		8	5			1
Soaring	2	4				

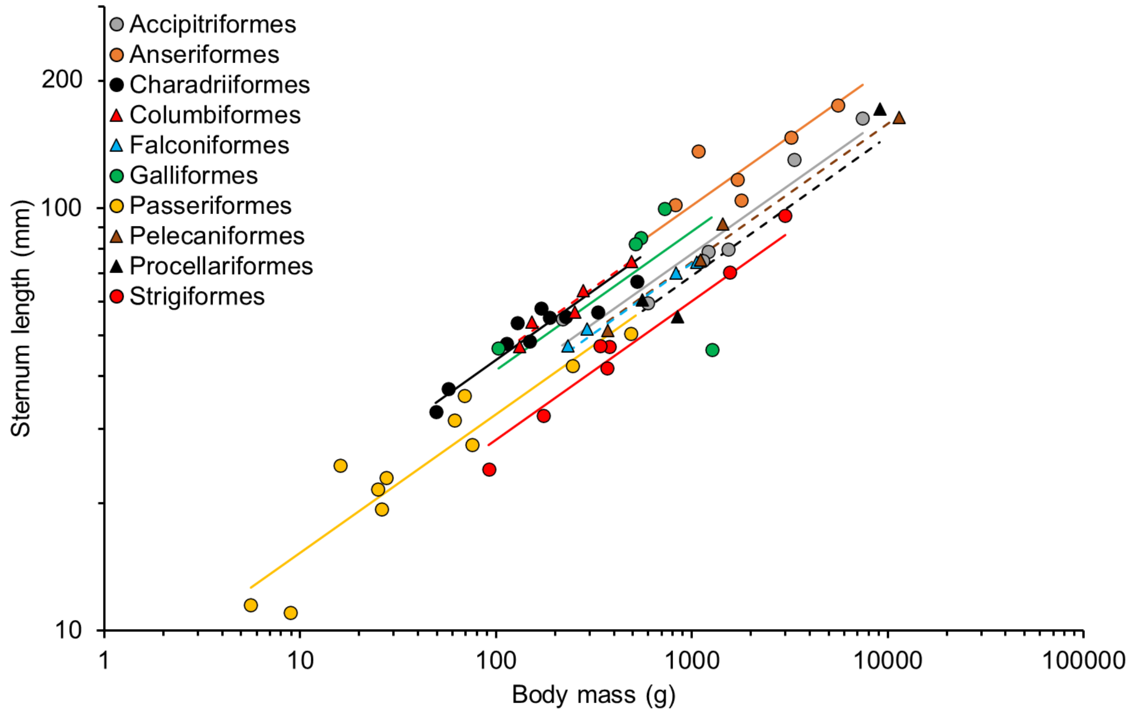

Figure S1. Relationship between body mass and sternum length for 10 orders. Lines indicate phylogenetically controlled relationships for each order (see text).

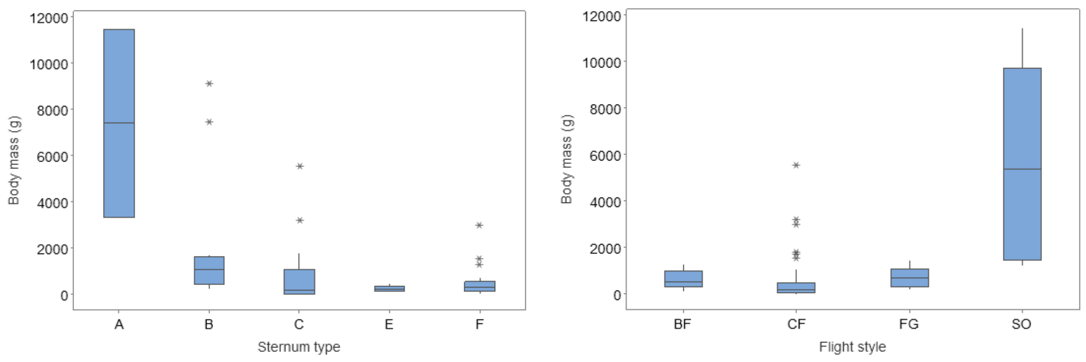

Figure S2. Boxplots showing the median, interquartile ranges and range of values for body mass for each sternum type (left panel) and flight style (right panel).

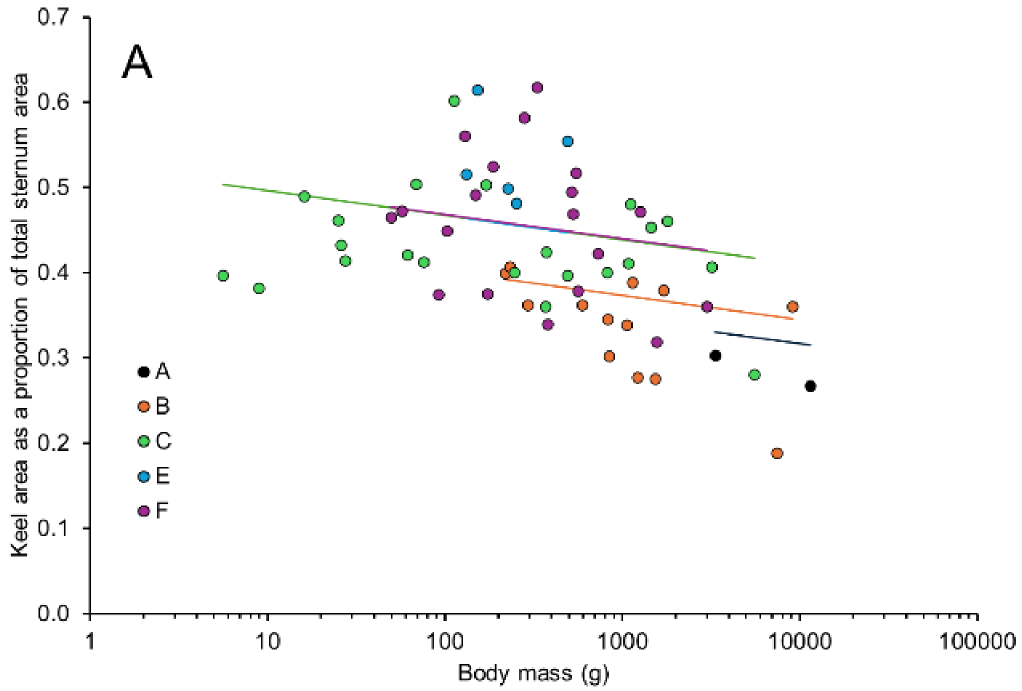

A
Keel area as a proportion of total sternum area
0.0
0.1
0.2
0.3
0.4
0.5
0.6
0.7
1
10
100
1000
10000
100000
Body mass (g)
A
B
C
E
F

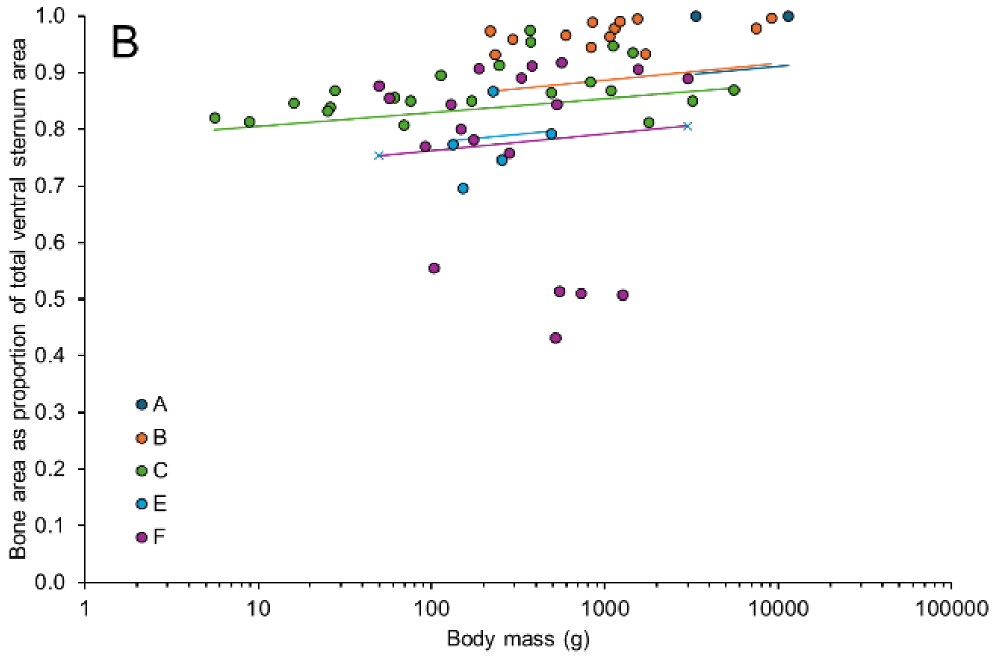

B
Bone area as proportion of total ventral sternum area
0.0
0.1
0.2
0.3
0.4
0.5
0.6
0.7
0.8
0.9
1.0
1
10
100
1000
10000
100000
Body mass (g)
A
B
C
E
F

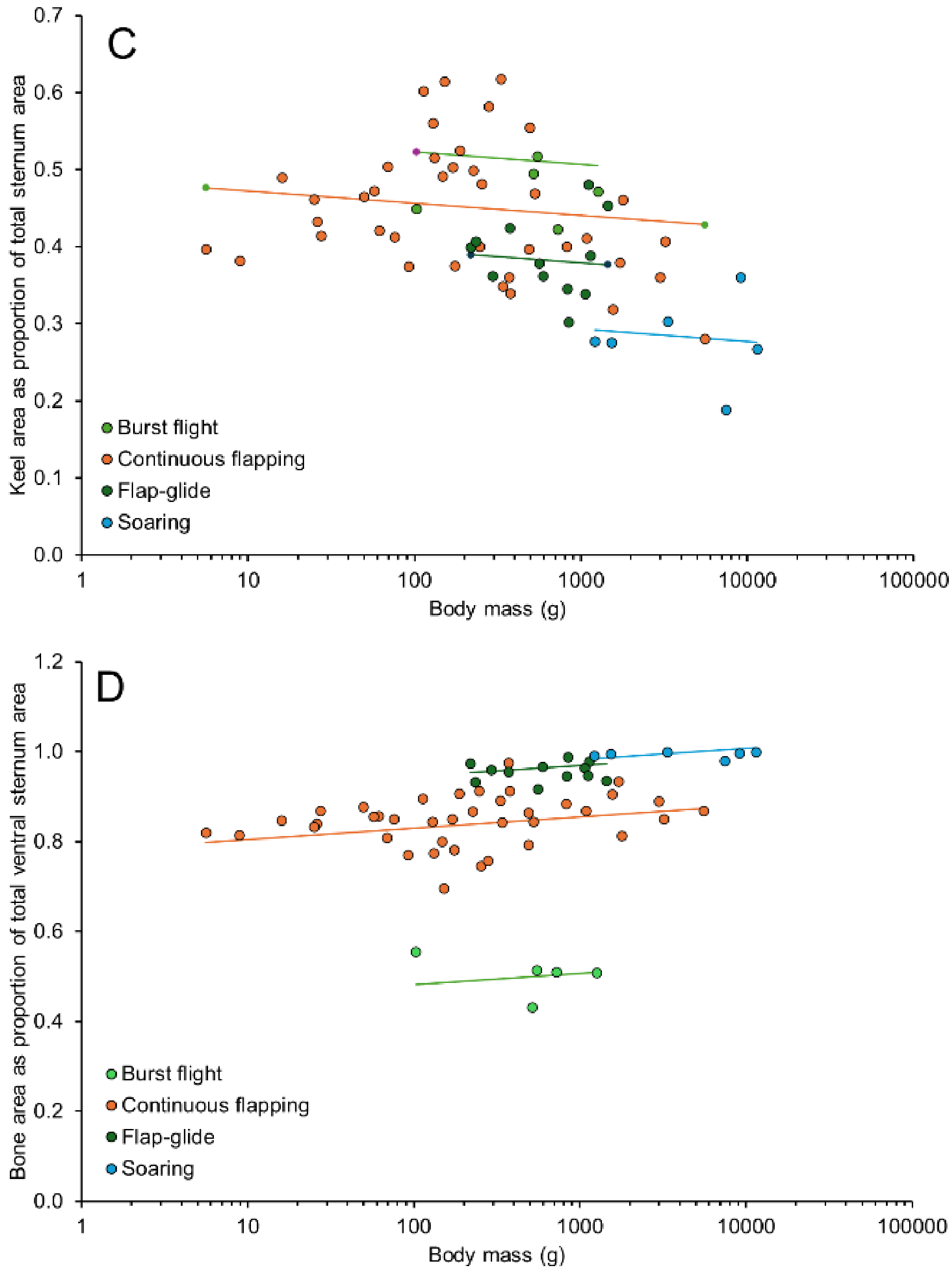

Figure S3. Relationships between body mass and (A) keel area as a proportion of the total sternum area (KA/TSA), and (B) the proportion of the ventral view of the sternum that is occupied by bone (PropBone) for five different sternum shapes, and body mass and (C) KA/TSA, and (D) Propbone for four flight styles. Lines indicate phylogenetically controlled relationships calculated in R for each order (see main text).
